# Effects of the COVID-19 Pandemic on the Lifestyle and Mental Health of Children in the Puducherry District: A Community-Based Cross-Sectional Study

**DOI:** 10.7759/cureus.63164

**Published:** 2024-06-25

**Authors:** Priskilla Johnson Jency, Raja Jeyapal Dinesh, Rajendran Dhanalakshmi, Adinarayanan Srividya, Palappurath Maliyakkal Azad, Ashwani Kumar

**Affiliations:** 1 Department of Epidemiology and Operational Research, Indian Council of Medical Research-Vector Control Research Centre, Puducherry, IND; 2 Department of Biostatistics and Vector-Borne Diseases Modeling, Indian Council of Medical Research-Vector Control Research Centre, Puducherry, IND; 3 Indian Council of Medical Research, Vector Control Research Centre, Puducherry, IND; 4 SIMATS, Saveetha University, Chennai, IND

**Keywords:** covid-19, strengths and difficulties questionnaire, pandemic, mental health, children

## Abstract

Introduction: The COVID-19 pandemic has tremendously disrupted societal behaviors and norms. People had to cope with new situations, including restrictions on free movement, home confinement, and school closures, among others. With less scope for physical classes, online classes became rampantly common during and after the pandemic. A virtual learning platform cannot replace the societal learning and preparation of children that normally occurs in school settings. The pandemic had a multifaceted impact on children, disrupting their routine work, social life, and mental health. Such uncertain circumstances are bound to interfere with their emotional well-being, with long-term consequences. It is imperative to screen for the effects of the pandemic situation among children for timely action.

Methods: A cross-sectional survey was carried out in both rural and urban areas of Puducherry, India, between February and April 2022, toward the fag end of the pandemic. Face-to-face interviews were conducted among caregivers of 621 children aged 6-17 years. Details such as sociodemographic, personal, and behavioral aspects of the child were collected. Emotional and behavioral difficulties during the pandemic were assessed using the parent (caregiver) version of the Strengths and Difficulties Questionnaire-25 (SDQ-25). Univariate analysis was performed using the chi-square test. Four different regression models were fitted to ascertain the factors influencing the overall difficulty score as well as the SDQ subscales, namely, the internalizing, externalizing, and prosocial scores. A P value of <0.05 was considered significant.

Results: Overall, 101 (16.3%) children aged 6-17 years were likely to have emotional and behavioral difficulties according to the SDQ scores. Abnormal externalizing, internalizing, and prosocial scores were documented among 160 (25.8%), 258 (41.5%), and 285 (45.9%) children, respectively. Caregivers reported disruptions in their children's academic performance (426, 68.6%), sleeping patterns (269, 43.3%), and eating habits (256, 41.2%). The use of digital devices for noneducational purposes was reported among 97 (35.9%) children. Younger caregivers (18-45 years), children who used digital devices for >2 hours per day, children who experienced any death due to COVID-19 in their family, and caregivers who perceived that the psychological changes in their children were due to the pandemic were predictors of abnormal SDQ scores. Physical activity for more than two hours per day reduced the risk of emotional and behavioral difficulties in children by 60%.

Conclusions: This research underscores the potential ramifications of the pandemic on the mental well-being and lifestyle of children. Implementing initiatives that promote positive mental health and conducting preventive screening for vulnerable populations, such as children, are considered essential, anticipating the challenges posed by such unprecedented pandemic circumstances in the future.

## Introduction

Since 2020, the COVID-19 pandemic has imposed a significant burden on cases and deaths globally [1]. Other facets of human well-being may also be affected, particularly the mental health of vulnerable groups such as children [2]. Stringent public health measures implemented during the pandemic, including complete lockdowns, school closures, and movement restrictions, may have had a negative impact on children's mental health. A change in daily routines, such as going to school, has been shown in several studies to potentially deprive children of their ability to develop social skills and emotional resilience [2-4]. The WHO speculated an increase in anxiety, depression, insomnia, and other emotional difficulties among children due to lack of social life and disrupted education [5]. Furthermore, unhealthy lifestyle practices such as reduced physical activity and increased screen time can adversely influence mental well-being [6,7].

Several studies have highlighted the psychological challenges children and adolescents in India have faced while coping with the pandemic's disruption of their daily lives [8-11]. A systematic review reported that the pandemic has led to increased anxiety, depression, and stress in children and adolescents [12]. Additionally, it has been observed that children are vulnerable to factors such as loneliness, fear of contracting or spreading the disease, mental trauma, loss of loved ones during the pandemic, domestic violence, family conflicts, and boredom proneness [13,14]. Even past pandemics (H1N1 pandemic in 2009) or crisis experiences globally have documented similar psychological effects on children [15]. It has been reported that around 119,170 children were orphaned due to COVID-19 with a rate of 0.3 per 1,000 children under 18 years of age. This bereavement of care would expose them to serious issues such as poverty, illiteracy, abuse, and mental trauma [16].

Thus, children's mental health is extremely important since it might affect them for the rest of their lives, even as adults. Younger minds are in the process of setting their personalities, which can be hampered by such interference and can lead to permanent cognitive or psychological effects [17]. At this juncture, it is critical to evaluate children's mental health from a public health perspective so that timely and appropriate support may be given. Furthermore, it helps in framing interventions that can be aimed at such vulnerable groups to face any similar crises in the future, if any. Hence, a community-based cross-sectional study was conducted to assess the psychological impact of the COVID-19 pandemic and its various associated factors, such as age, gender, socioeconomic status, use of digital devices, physical activity, academic performance, and online classes, on children aged 6-17 years in the district of Puducherry.

## Materials and methods

Study design and settings

**Figure 1:**
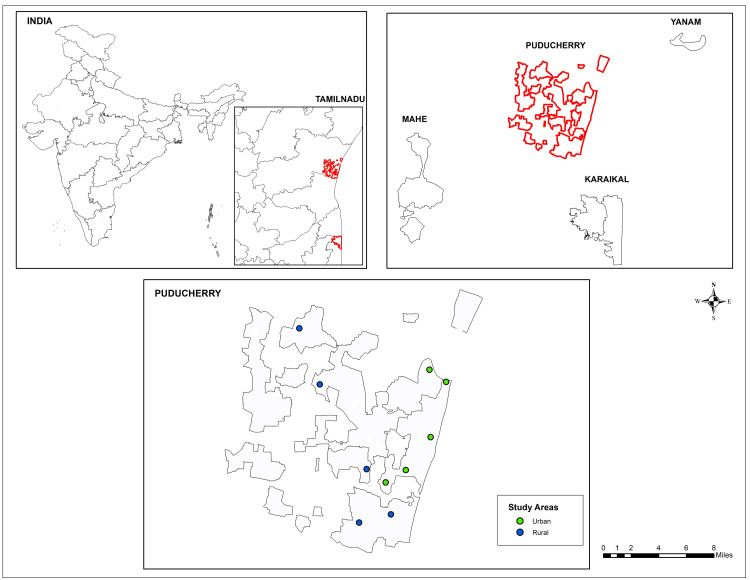
Study areas in Puducherry, India

The cross-sectional study was conducted in the rural and urban areas of Puducherry, a coastal town on the Coromandel Coast of India, from February 2022 to April 2022 (Figure 1). According to the census 2011, Puducherry has a population of 12.3 lakhs, of whom 3.5 lakhs are children [18].

During the early phase of COVID-19 in 2020, the local administration stringently implemented lockdowns with movement restrictions; the closure of schools, colleges, other educational institutions, and nonessential workplaces; and a ban on social gatherings, among several others, as preventive measures to contain the pandemic. In 2022, Puducherry was in the phase of re-establishing routine services and practices in a stepwise manner. Although there was no complete lockdown, pandemic-based movement restrictions and partial school closures were in place owing to the resurgence of new COVID-19 variants such as omicron [19]. Many schools resorted to teaching classes online, especially primary classes, a new norm that came into existence after the pandemic.

Sample size

A minimum sample size of 300 was calculated considering 34% COVID-19-related psychological impact among children [20], allowing for a 7% margin of error (absolute error) at the 5% level of significance, a nonresponse rate of 10%, and a design effect of 1.5 (considering the clustering effect due to homogeneity in responses). Therefore, 300 children from both rural and urban areas of Puducherry were enrolled in the study.

Sampling strategy

**Figure 2:**
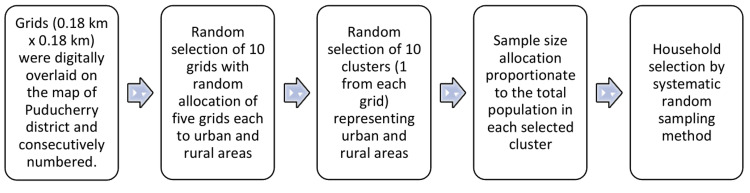
Sampling strategy

Ten clusters (villages or wards) were chosen using the grid sampling method, five of which represented the Puducherry district's rural and urban areas. The detailed sampling strategy is described in the flowchart (Figure 2). A total of 505 households were systematically selected to meet the desired sample size. If any of the selected households were locked or children were not available, the next adjacent household with children was included until the target sample size was achieved.

Study tools

The survey questionnaire consisted of two parts. The first part included general details pertaining to sociodemographics, exposure to COVID-19, and a child's lifestyle and behavioral aspects (eating, sleeping, screen time, education, recreation, and social interaction). The second part included the parent (caregiver) version of the Strengths and Difficulties Questionnaire-25 (SDQ-25) [21]. The SDQ measures negative and positive behavioral attributes using 25 items primarily focused on five domains: emotional symptoms, hyperactivity (or) inattention, conduct problems, peer relationship problems, and prosocial behavior. The validated tool is available in the local language Tamil and is free to access and use for research purposes without any modifications [22]. The SDQ scale items are rated on a 3-point scale: "certainly true," "somewhat true," or "not true." The sum of the four (excluding prosocial) domains generates a total difficulty score ranging from 0 to 40. There are three subscale domains: (1) the internalizing score (sum of emotional and peer problems), (2) the externalizing score (sum of conduct and hyperactivity), and (3) the prosocial score. Higher scores indicate a possible psychological impact, except on the prosocial scale, where lower scores indicate a likely psychological impact. The two amalgamated scales, internalizing and externalizing scores, are commonly used for categorizing difficulty scores in community samples [23]. The cutoff levels for the overall score and for each subscale are depicted in Table 1.

**Table 1:** SDQ-25 cutoff scores SDQ-25: Strengths and Difficulties Questionnaire-25

SDQ score (prosocial score)	Normal	Slightly raised (lowered)	High (low)	Very high (very low)
Total difficulties score	0-13	14-16	17-19	20-40
Internalizing score	0-3	4-7	8-10	11-20
Externalizing score	0-7	8-10	11-13	14-20
Prosocial score	8-10	7	6	0-5

Study subjects and data collection

The study participants were children aged between six and 17 years. Children who resided in Puducherry for less than one year or were already diagnosed with any mental health disorders were excluded. The questionnaire was administered to the caregivers (either a parent or any adult family member involved in childcare) available at the time of the survey. Data were collected by a medico-social worker and a medical specialist. Both field investigators received training from a consulting psychiatrist on how to administer the SDQ correctly in the community. The social worker explained the study in detail to all participants and assured them that their identities would be kept anonymous and that the data would only be utilized for research. Only those children and caregivers who were physically available and willing to participate were included. Written informed consent (from caregivers) and verbal or written assent (from children aged 7-17 years) were obtained prior to the personal interview. Children with likely difficulties as assessed by SDQ scores were referred for counselling with a psychiatrist.

Statistical analysis

The Statistical Package for the Social Sciences version 21.0 (IBM SPSS Statistics, Armonk, NY) was used for the data analysis. Continuous variables such as age are expressed as mean ± standard deviation (SD). Categorical variables are summarized as frequencies and percentages. The overall SDQ scores and the three subscale scores were dichotomized as normal or abnormal (all categories other than normal) for further analysis. The associations between abnormal SDQ scores and internalizing, externalizing, and prosocial domain scores and various independent variables were assessed using the chi-square test. Variables with P < 0.20 were identified and tested for collinearity. Finally, four regression models were fitted to identify the significant predictors for the overall abnormal SDQ scores and the three subscale scores. P < 0.05 was considered significant.

## Results

Sociodemographic details

Overall, 505 caregivers (from 505 households) reported on the mental health of 621 children aged 6-17 years of either gender from the rural and urban areas of Puducherry. Most children hail from nuclear families. More than 80% of the caregivers were female, with a mean age of 38.5 ± 8.9 years. The sociodemographic characteristics of the participants are summarized in Table 2.

**Table 2:** Sociodemographic characteristics of the study participants (N = 621) *Modified BG Prasad's scale of socioeconomic status (2022) [24]

Sociodemographic details		Number (%)
Area	Urban	306 (49.3)
	Rural	315 (50.7)
Child age (years)	6-10	291 (46.9)
	11-17	330 (53.1)
Child gender	Male	320 (51.5)
	Female	301 (48.5)
Caregiver age (years) (n = 505)	18-45	418 (82.8)
	>45	87 (17.2)
Caregiver gender (n = 505)	Male	63 (12.5)
	Female	442 (87.5)
Family type	Nuclear	415 (66.8)
	Joint	206 (33.2)
*Socioeconomic status	Class I-II	440 (70.8)
	Class III-V	181 (29.2)
School type	Government	322 (51.8)
	Private	299 (48.2)
Number of children	Single	55 (8.9)
	≥2	566 (91.1)

Education during the pandemic

The study children, whose mean age was 11.0 ± 3.1 years, studied in either government or private schools. During the pandemic, 382 (61.5%) children reported access to online classes. A total of 266 (77.1%) private school students and 116 (42%) students from government schools, respectively, had access to online classes (P < 0.001). A significant difference (P < 0.001) was also observed among children from 210 (68.6%) urban and 172 (54.6%) rural areas in terms of access to online classes. Caregivers reported that 354 (57%) children attended online classes during the pandemic (Table 3). Comparatively, a greater proportion of students from urban areas (203, 66.3%) and private schools (261, 75.7%) attended online classes than did students from rural areas (151, 47.9%) and government schools (93, 33.7%). The difference was statistically significant (P < 0.001). Children who did not have access to online classes either resorted to self-reading with available books (92, 38.5%), learned with the help of friends and relatives (38, 15.9%), or did not pursue learning at all (109, 45.6%) during the pandemic period.

**Table 3:** Association of abnormal SDQ scores with independent variables (N = 621) *Significant at the 5% level SDQ: Strengths and Difficulties Questionnaire, uOR: unadjusted odds ratio, aOR: adjusted odds ratio, 95% CI: 95% confidence interval, COVID-19: coronavirus disease 2019

Independent variables		Number	Abnormal SDQ scores (number (%))	Univariate analysis		Multivariate analysis	
				uOR (95% CI)	P value	aOR (95% CI)	*P value
Child gender	Male	320	60 (18.8)	1	0.083	1	0.107
	Female	301	41 (13.6)	0.7 (0.4-1.1)		0.6 (0.4-1.1)	
Caregiver age (years) (n = 505)	>45	87	10 (11.5)	1	0.074	1	0.047*
	18-45	418	91 (21.8)	2.0 (0.9-4.4)		2.7 (1.1-6.8)	
Caregiver gender (n = 505)	Male	63	9 (14.3)	1	0.038	1	0.093
	Female	442	95 (21.5)	3.0 (1.1-8.5)		2.5 (0.8-7.8)	
Family type	Nuclear	415	61 (14.7)	1	0.134	1	0.537
	Joint	206	40 (19.4)	1.4 (0.9-2.2)		1.2 (0.7-2.1)	
Socioeconomic status	Class I-II	440	77 (17.5)	1	0.193	1	0.198
	Class III-V	181	24 (13.3)	0.7 (0.4-1.2)		0.6 (0.3-1.3)	
Attend online classes	Did not attend	267	52 (19.5)	1	0.061	1	0.264
	Attended	354	49 (13.8)	0.7 (0.4-1.0)		0.6 (0.3-1.4)	
Access to online classes	Yes	382	56 (14.7)	1	0.171	1	0.580
	No	239	45 (18.8)	1.4 (0.9-2.1)		1.2 (0.6-2.6)	
Digital device usage	Did not use	351	48 (13.7)	1	0.003	1	0.030
	≤2 hours	173	26 (15)	1.1 (0.7-1.9)	0.676	1.5 (0.8-2.9)	0.210
	>2 hours	97	27 (27.8)	2.4 (1.4-4.2)	<0.001	2.6 (1.3-5.4)	0.009*
Physical activity	No activity	136	29 (21.3)	1	0.036	1	0.138
	≤2 hours	351	59 (16.8)	0.8 (0.5-1.2)	0.246	0.7 (0.4-1.4)	0.363
	>2 hours	134	13 (9.7)	0.4 (0.2-0.8)	0.010	0.4 (0.2-0.9)	0.047*
Death due to COVID-19 in the family	Yes	9	4 (44.4)	1	0.03	1	0.016*
	No	612	97 (15.8)	0.2 (0.1-0.9)		0.06(0.01-0.6)	
Eating habits	Not affected	365	52 (14.2)	1	0.104	1	0.877
	Affected	256	49 (19.1)	1.4 (0.9-2.2)		0.9 (0.4-1.9)	
Sleeping habits	Not affected	352	45 (12.8)	1	0.007	1	0.657
	Affected	269	56 (20.8)	1.8 (1.2-2.8)		1.2 (0.6-2.3)	
Academic performance	Not affected	195	16 (8.2)	1	<0.001	1	0.170
	Affected	426	85 (20)	2.8 (1.6-4.9)		1.6 (0.8-3.3)	
Recreation	Not affected	533	80 (15)	1	0.037	1	0.153
	Affected	88	21 (23.9)	1.8 (1.0-3.1)		0.5 (0.2-1.3)	
Social interaction	Not affected	584	86 (14.7)	1	<0.001	1	0.067
	Affected	37	15 (40.5)	4.0 (2.0-7.9)		2.5 (0.9-6.6)	
Psychological changes were due to the pandemic	No	409	40 (9.8)	1	<0.001	1	<0.001*
	Yes	212	61 (28.8)	3.7 (2.4-5.8)		3.0 (1.7-5.4)	

Lifestyle changes during the pandemic

Caregivers reported notable changes in their child's lifestyle or daily routine. Most of the caregivers (426, 68.6%) felt that the academic performance of their children was affected. More than two-fifths of the respondents opined that it affected their children's sleep (269, 43.3%) and eating habits (256, 41.2%). Only 88 (14.2%) and 37 (6%) caregivers responded that the pandemic had affected their children's recreational and social activities, respectively. Additionally, 270 (43.5%) children used digital devices such as mobile phones or laptops for attending online classes. Among those who used digital devices, 97 (35.9%) used them for more than two hours a day (Table 3). Caregivers reported that 193 (31.1%) children spent time watching television. Nearly half (286, 46.1%) of the children had learned a new hobby or skill, such as drawing, painting, or cooking, during the pandemic period.

Caregivers reported post-pandemic emotional and behavioral difficulties in children

The caregiver-reported SDQ (based on the overall score) showed that 101 (16.3%) children aged 6-17 years were likely to have emotional and behavioral difficulties. More than a quarter (212, 34.1%) of caregivers ascribed significant psychological changes among children to the COVID-19 pandemic (Table 3). Although 53 (18.2%) children aged 6-10 years and 48 (14.5%) adolescents aged 11-17 years had abnormal SDQ scores, the difference was not statistically significant (P = 0.217). Only 37 (5.9%) were aware of mental health services such as teleconsultation and helplines provided by the government; however, none reported having availed any of these facilities in the past.

**Figure 3:**
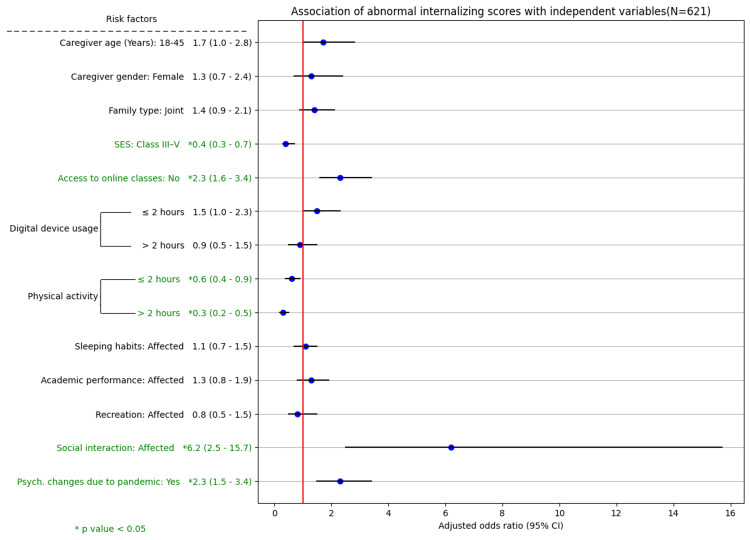
Association of abnormal internalizing scores with independent variables (N = 621) SES: socioeconomic status, 95% CI: 95% confidence interval

**Figure 4:**
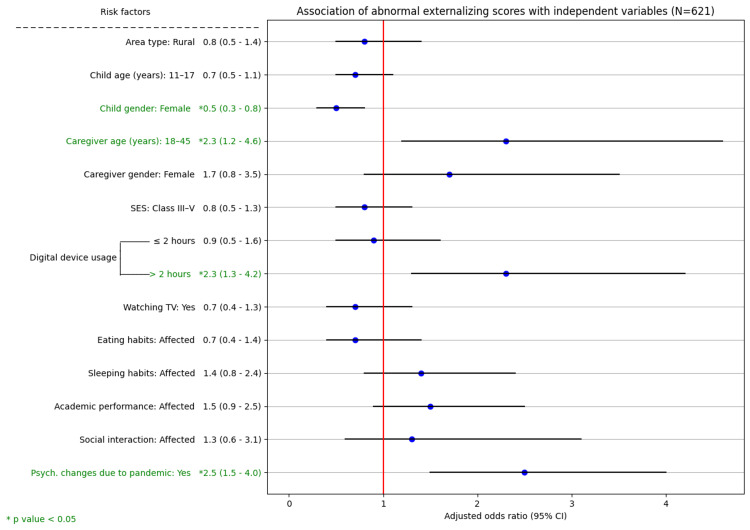
Association of abnormal externalizing scores with independent variables (N = 621) SES: socioeconomic status, 95% CI: 95% confidence interval

**Figure 5:**
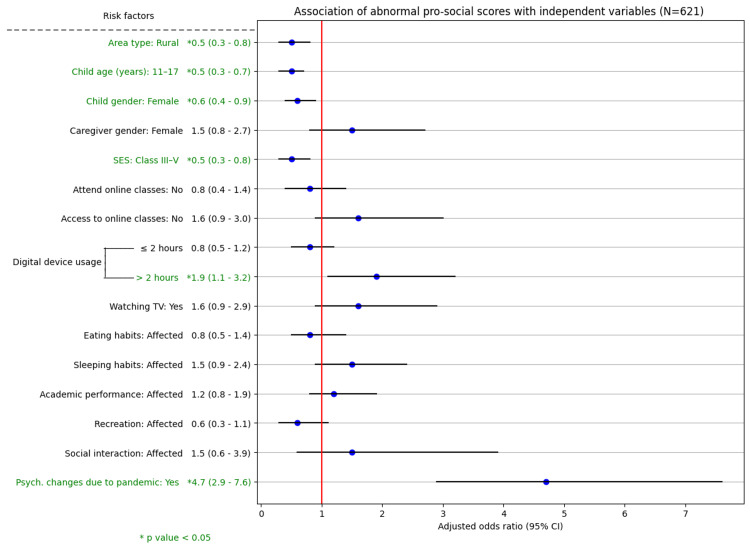
Association of abnormal prosocial scores with independent variables (N = 621) SES: socioeconomic status, 95% CI: 95% confidence interval

On further categorization, 160 (25.8%) had abnormal externalizing scores, suggesting likely conduct and hyperactivity problems; 258 (41.5%) had abnormal internalizing scores, suggesting likely emotional and peer problems. Overall, 285 (45.9%) children had abnormal prosocial scores, suggesting problems with socialization with others. Compared with adolescents, children aged 6-10 years had abnormal externalizing (P = 0.010) and prosocial (P < 0.001) scores (Figures 3-5).

On univariate analysis, caregivers' gender, use of digital devices, physical activity, death due to COVID-19 in the family, perception of sleeping habits, academic performance, recreational activities, social interaction, and perceptions that the psychological changes in the child were due to the pandemic were found to be significantly associated with abnormal SDQ scores (P < 0.05). According to multivariate logistic regression analysis, caregivers aged 18-45 years reported 2.7 times more abnormal SDQ scores than those aged >45 years. Compared with their counterparts, 60% and 94% of the children who used digital devices for >2 hours a day and who experienced death in the family due to COVID-19, respectively, were more likely to have abnormal SDQ scores. Children who engaged in physical activity for >2 hours a day were 60% less likely to have emotional and behavioral difficulties (P = 0.047). The caregivers' perception that the psychological changes in the children were due to the COVID-19 pandemic was a significant predictor of abnormal SDQ scores (Table 3).

Determinants of internalizing, externalizing, and prosocial SDQ scores

The associations of various independent variables with the subscales of the SDQ, namely, the internalizing, externalizing, and prosocial scores, were examined using the chi-square test. Variables such as academic performance, social interaction, and the perception that psychological changes were due to COVID-19 were significantly associated with abnormal scores across all three subscales. On multivariate logistic regression analysis, socioeconomic class, access to online classes, physical activity, and a child's social interaction were the determinants of abnormal internalizing scores. The child's gender, caregiver age, and use of digital devices were the determinants of abnormal externalizing scores. Area type, child age, child gender, socioeconomic class, and use of digital devices were the determinants of abnormal prosocial scores. Caregivers' perception that the psychological changes in children were due to the COVID-19 pandemic was a significant predictor of abnormal scores across all three SDQ subscales (P < 0.001) (Figures 3-5).

## Discussion

In the present study, 101 (16.3%) of Puducherry children aged 6-17 years were found to have behavioral and emotional difficulties. Studies from northern and northeastern India have reported 12.2%-23.1% behavioral and emotional difficulty among children based on the SDQ and other evaluation scales [9-11,25]. These studies were conducted in an online mode during the peak of the pandemic and involved only those with access to internet services. Subscale analysis of the SDQ scores revealed that 258 (41.5%), 160 (25.8%), and 285 (45.9%) children had abnormal internalizing, externalizing, and prosocial scores, respectively. The subscales reported a much greater proportion of children with psychological difficulties than did the overall SDQ. Internalizing, externalizing, and prosocial difficulties as high as 66.5%, 36.8%, and 57.4%, respectively, have been reported among children elsewhere [25].

The study revealed that children belonging to middle to lower socioeconomic classes had fewer peer and emotional difficulties than those in upper socioeconomic classes (P = 0.005). Females, children from rural areas, adolescents, and those from middle to lower socioeconomic classes have higher prosocial scores than their counterparts, reflecting better socialization. These findings are contrary to those from England and India [20,26]. This may be because younger children, being in their early developmental stages, are highly vulnerable to mental disturbances owing to their altered routines, especially when forced to stay in confinement [27]. Additionally, children from urban and upper social classes are prone to experiencing psychological stress due to factors such as minimal contact with peers, more noncontact leisure activities, and witnessing family conflicts [4,6,13]. Younger caregivers (18-45 years) reported greater emotional and behavioral difficulties among children, which is consistent with the findings of other studies [28]. It is likely that these younger caregivers were more apprehensive about their children than were the older caregivers.

Screen time was found to be a double-edged sword in our study. Children who had access to online classes had lower internalizing scores. However, children who used digital devices for more than two hours per day for noneducational purposes were more likely to have emotional and behavioral difficulties. Although digital devices help children stay connected with friends and family and continue their education, excessive screen time has been attributed to negative mental health outcomes such as anxiety, depression, and poor quality of sleep [7,29]. Cyberbullying and exposure to inappropriate content are additional concerns related to digital device usage in children, although they are not explicitly captured in this study [7,29].

Caregivers reported marked changes in the lifestyle and daily routine of their children in aspects such as academic performance (68.6%), sleeping patterns (43.3%), and eating habits (41.2%), although these changes were not found to be significant. Regular physical activity was protective against developing emotional and behavioral difficulties, which corroborates the findings of other studies [30]. Although the pandemic confined children to their homes, it provided an opportunity to inculcate productive hobbies (46.1%) and spend quality time with family members, as documented in the literature [20].

Children who experienced the death of a family member due to the pandemic were significantly at greater odds of developing behavioral and emotional difficulties. It is well known that the loss of a family member due to a pandemic can amplify psychological distress [14]. The caregivers' perception that the pandemic has affected their children's lifestyle and psychological well-being was a significant predictor across overall and domain-wise SDQ scores. This strongly suggests the need for developing strategies to screen for and address psychological disturbances among children, at least during the post-pandemic period [31,32].

It is imperative to implement efficient approaches to mitigate the psychological disturbances induced by pandemic-like situations in the future. This includes highlighting positive aspects of preventive measures, regulating digital device usage, encouraging positive coping strategies, and building resilience among younger age groups [20,27,29-31]. The National Mental Health Programme and Centers of Excellence, such as the National Institute of Mental Health and Neurosciences (NIMHANS), established 24/7 helpline services and several outreach measures during the pandemic [33]. Similarly, mental health services have been included in the existing public health services through the District Mental Health Programme (DMHP) [34]. Even in Puducherry, the DMHP is well established for catering to the mental health needs of the community at all levels of healthcare. However, in the present study, only 5.9% were aware that such services existed, and none of them ever availed them. These findings warrant extensive awareness campaigns, especially at schools and colleges, on mental health and reducing the associated stigma. Parents and guardians should also be considerate toward their children during such crisis times and create a conducive environment for their mental well-being [32,35].

Strengths and limitations

This cross-sectional study conducted in the Puducherry district is the first to capture the influence of the pandemic on the psychological status of children in the region. As data were collected through face-to-face interviews by trained investigators from different areas selected using the grid sampling method, they have higher quality with good completion rates and reliability compared to studies conducted in the online mode. Additionally, the study participants with high SDQ scores were recommended to visit a psychiatrist, although their status was not compared with that of a clinical diagnosis. Furthermore, the participants were not followed up owing to the study design and time constraints. Since it is a cross-sectional survey with no baseline data, it is difficult to prove the causal relationship warranting longitudinal comparative studies in the future.

## Conclusions

Our study indicates that one in every six children in Puducherry has possible emotional and behavioral difficulties post-pandemic. The dependency and reliance on digital devices have considerably increased since the pandemic, and it is crucial to prioritize the management of its usage among children. Furthermore, to reduce the risk of overt psychological problems in children, it is necessary to foster positive social interaction patterns both at home and in the school environment. It is desirable to conduct campaigns to promote positive mental health behaviors and screen vulnerable populations on a regular basis. Initiating community-based screening for mental issues is not a goal on its own; the detection of child psychiatric disorders is not an end in itself. More research is necessary to ascertain whether enhanced or earlier detection results in superior outcomes. These findings may help address mental health issues efficiently during such public health emergencies in the future.

## References

[1] (2024). World Health Organization: Coronavirus disease (COVID-19) pandemic. https://www.who.int/emergencies/diseases/novel-coronavirus-2019.

[2] Tso WW, Wong RS, Tung KT (2022). Vulnerability and resilience in children during the COVID-19 pandemic. Eur Child Adolesc Psychiatry.

[3] Chaabane S, Doraiswamy S, Chaabna K, Mamtani R, Cheema S (2021). The impact of COVID-19 school closure on child and adolescent health: a rapid systematic review. Children (Basel).

[4] Rajmil L, Hjern A, Boran P, Gunnlaugsson G, Kraus de Camargo O, Raman S (2021). Impact of lockdown and school closure on children's health and well-being during the first wave of COVID-19: a narrative review. BMJ Paediatr Open.

[5] (2024). World Health Organization: Mental health and COVID-19. https://www.who.int/europe/emergencies/situations/covid-19/mental-health-and-covid-19.

[6] Liu Q, Zhou Y, Xie X (2021). The prevalence of behavioral problems among school-aged children in home quarantine during the COVID-19 pandemic in China. J Affect Disord.

[7] Twenge JM, Campbell WK (2018). Associations between screen time and lower psychological well-being among children and adolescents: evidence from a population-based study. Prev Med Rep.

[8] Panda PK, Gupta J, Chowdhury SR (2021). Psychological and behavioral impact of lockdown and quarantine measures for COVID-19 pandemic on children, adolescents and caregivers: a systematic review and meta-analysis. J Trop Pediatr.

[9] Shukla M, Pandey R, Singh T, Riddleston L, Hutchinson T, Kumari V, Lau JY (2021). The effect of COVID-19 and related lockdown phases on young peoples’ worries and emotions: novel data from India. Front Public Health.

[10] Sama BK, Kaur P, Thind PS, Verma MK, Kaur M, Singh DD (2021). Implications of COVID-19-induced nationwide lockdown on children's behaviour in Punjab, India. Child Care Health Dev.

[11] Saurabh K, Ranjan S (2020). Compliance and psychological impact of quarantine in children and adolescents due to COVID-19 pandemic. Indian J Pediatr.

[12] Chawla N, Tom A, Sen MS, Sagar R (2021). Psychological impact of COVID-19 on children and adolescents: a systematic review. Indian J Psychol Med.

[13] Bhatia A, Fabbri C, Cerna-Turoff I (2021). Violence against children during the COVID-19 pandemic. Bull World Health Organ.

[14] Joaquim RM, Pinto AL, Guatimosim RF (2021). Bereavement and psychological distress during COVID-19 pandemics: the impact of death experience on mental health. Curr Res Behav Sci.

[15] Sprang G, Silman M (2013). Posttraumatic stress disorder in parents and youth after health-related disasters. Disaster Med Public Health Prep.

[16] Hillis SD, Unwin HJ, Chen Y (2021). Global minimum estimates of children affected by COVID-19-associated orphanhood and deaths of caregivers: a modelling study. Lancet.

[17] Racine N, Korczak DJ, Madigan S (2022). Evidence suggests children are being left behind in COVID-19 mental health research. Eur Child Adolesc Psychiatry.

[18] (2024). National Institute of Public Cooperation and Child Development: Statistics on children in India. https://www.nipccd.nic.in/file/reports/handbk18.pdf.

[19] (2024). Puducherry district: Corona COVID-19 (New). http://dt.gov.in/document-category/corona-covid-19-new.

[20] (2024). Child Rights and You: Impact of COVID-19 pandemic on children. https://www.cry.org/downloads/safety-and-protection/Impact-of-COVID19-pandemic-on-children.pdf.

[21] Goodman R (1997). The Strengths and Difficulties Questionnaire: a research note. J Child Psychol Psychiatry.

[22] Lukumar P, Wijewardana K, Hermansson J, Lindmark G (2008). Validity and reliability of Tamil version of Strengths and Difficulties Questionnaire self-report. Ceylon Med J.

[23] Goodman A, Lamping DL, Ploubidis GB (2010). When to use broader internalising and externalising subscales instead of the hypothesised five subscales on the Strengths and Difficulties Questionnaire (SDQ): data from British parents, teachers and children. J Abnorm Child Psychol.

[24] Sharma R (2013). Revision of Prasad's social classification and provision of an online tool for real-time updating. South Asian J Cancer.

[25] Nath S, Gogoi V, Linganna SB, Baruah J, Sutradhar B (2022). Behavioural and emotional difficulties in school children during COVID 19 pandemic using narrowband dimensions of SDQ: online survey from North? East India. Ind Psychiatry J.

[26] Panagi L, White SR, Pinto Pereira SM (2024). Mental health in the COVID-19 pandemic: a longitudinal analysis of the CLoCk cohort study. PLoS Med.

[27] Wissmath B, Mast FW, Kraus F, Weibel D (2021). Understanding the psychological impact of the COVID-19 pandemic and containment measures: an empirical model of stress. PLoS One.

[28] Rathgeb C, Schillok H, Voss S (2022). Emotional situation of children and adolescents during the COVID-19 pandemic in Germany: results from the COVID-19 Snapshot Monitoring (COSMO). Int J Environ Res Public Health.

[29] Prime H, Wade M, Browne DT (2020). Risk and resilience in family well-being during the COVID-19 pandemic. Am Psychol.

[30] Dienlin T, Johannes N (2020). The impact of digital technology use on adolescent well-being. Dialogues Clin Neurosci.

[31] Kopp PM, Möhler E, Gröpel P (2024). Physical activity and mental health in school-aged children: a prospective two-wave study during the easing of the COVID-19 restrictions. Child Adolesc Psychiatry Ment Health.

[32] Wang G, Zhang Y, Zhao J, Zhang J, Jiang F (2020). Mitigate the effects of home confinement on children during the COVID-19 outbreak. Lancet.

[33] (2024). NIMHANS: COVID-19 information resources. https://nimhans.ac.in/health-information-nimhans/covid19-information/.

[34] Singh OP (2018). District Mental Health Program - need to look into strategies in the era of Mental Health Care Act, 2017 and moving beyond Bellary model. Indian J Psychiatry.

[35] Fegert JM, Vitiello B, Plener PL, Clemens V (2020). Challenges and burden of the coronavirus 2019 (COVID-19) pandemic for child and adolescent mental health: a narrative review to highlight clinical and research needs in the acute phase and the long return to normality. Child Adolesc Psychiatry Ment Health.

